# Simple Random Sampling-Based Probe Station Selection for Fault Detection in Wireless Sensor Networks

**DOI:** 10.3390/s110303117

**Published:** 2011-03-14

**Authors:** Rimao Huang, Xuesong Qiu, Lanlan Rui

**Affiliations:** 1 State Key Laboratory of Networking and Switching Technology, Beijing University of Posts and Telecommunications, Beijing 100876, China; E-Mails: xsqiu@bupt.edu.cn (X.Q.); llrui@bupt.edu.cn (L.R.); 2 Department of Computer and Information Science, Fujian Agriculture and Forestry University, Fuzhou 350002, China

**Keywords:** simple random sampling, probe station, fault detection, wireless sensor network, probing frequency, Pareto principle

## Abstract

Fault detection for wireless sensor networks (WSNs) has been studied intensively in recent years. Most existing works statically choose the manager nodes as probe stations and probe the network at a fixed frequency. This straightforward solution leads however to several deficiencies. Firstly, by only assigning the fault detection task to the manager node the whole network is out of balance, and this quickly overloads the already heavily burdened manager node, which in turn ultimately shortens the lifetime of the whole network. Secondly, probing with a fixed frequency often generates too much useless network traffic, which results in a waste of the limited network energy. Thirdly, the traditional algorithm for choosing a probing node is too complicated to be used in energy-critical wireless sensor networks. In this paper, we study the distribution characters of the fault nodes in wireless sensor networks, validate the Pareto principle that a small number of clusters contain most of the faults. We then present a Simple Random Sampling-based algorithm to dynamic choose sensor nodes as probe stations. A dynamic adjusting rule for probing frequency is also proposed to reduce the number of useless probing packets. The simulation experiments demonstrate that the algorithm and adjusting rule we present can effectively prolong the lifetime of a wireless sensor network without decreasing the fault detected rate.

## 1. Introduction

A wireless sensor network usually consists of a potentially large number of low-cost, low-power nodes, which contain sensing, data processing, and communicating components. The applications of wireless sensor networks are very wide, such as environmental monitoring, public safety, medicine, the military, and numerous other fields. In these situations, nodes are often deployed in complex and extreme environments, exposed to high temperatures, high humidity and so on. Consequently, the sensor nodes have higher fault rates than traditional network nodes [1], and the maintenance and replacement of components will often be prohibitively expensive. These features pose some challenges to fault management to help a wireless sensor network to achieve its intended purpose. Surveys of the topic of wireless sensor networks fault management can be found in [2,3].

A complete fault management task can be divided into three sequential phases, *i.e.*, fault detection, fault diagnosis and fault recovery. Fault detection is the first phase of fault management, where an unexpected failure should be properly identified by the network system.

We can divide all existing fault detection approaches into active and passive detection. Active detection is an active, effective, and adaptive network detection technique, which can detect and localize the faults in the network as soon as possible by sending out probing packets, which include some measurement parameters, into the network. In contrast, passive detection only analyzes the messages already present to infer the existence of network faults without sending out additional probing packets. In this study, we mainly discuss active detection.

In a wireless sensor network, a probe station for fault detection is a sensor node used to gather information about the status of nodes by sending out probing packets and receiving feedback messages. In most active detection approaches, whether the centralized approach [4,5], neighbor coordination approach [6–9], or cluster-based approach [10,11], some overhead is imposed upon the network because of these additional probing packets. Most early work on this topic treated the manager nodes as default probe stations, but the manager nodes are already burdened with great overhead, so any additional fault detection tasks would make the busy manager nodes fail more quickly.

Furthermore, most wireless sensor networks do not have any fault nodes at the beginning of their activity. Any probing packets sent out during this time would thus not find any fault information. These useless probing packets will result in some wasted energy, a problem that should be avoided in energy limited wireless sensor networks.

In addition, the wireless sensor network could change over time. Some nodes may get damaged due to the unpredictable factors, some nodes may run out of power and some nodes may cease communication. In this situation, choosing fixed nodes as probe stations is not a good solution and therefore, care should be paid to the selection of the number of probe stations, the locations of probe stations and the probing frequency for active detection.

Some complex approaches have been proposed in [12–18] however they are applied to traditional computer networks, and do not take consideration the most important feature of WSNs, namely the fact that resources are limited. Additionally, in the large-size network environment, complex computing tasks often consume a lot of time, which can result in fact that the status computed by the probe stations may not reflect the current network state.

The goals of this study are: (1) the dynamic selection of appropriate sensor nodes as probe stations as soon as possible; (2) the reduction of the useless probing packets. Before explaining the solution we propose, we first introduce two important principles:

- The placement of sensors can vary significantly in different applications. In a “structured” sensor network application (e.g., an intelligent building system), the position of sensor nodes are pre-determined, whereas in an “unstructured” application (e.g., battlefield surveillance), the sensor nodes are randomly distributed in the monitoring field. In this work, we focus on the latter case where the sensor nodes are randomly deployed, and the locations of sensors can be modeled by a stationary two-dimensional Poisson point process [19].
- Pareto principle: It is widely believed that a small number of modules in any system are likely to contain the majority of the total system faults [20]. This is often referred to as the ‘20–80 rule’ in the sense that 80% of the faults are contained in 20% of the modules.

Our contribution lies in that we first propose a Simple Random Sampling based selecting (SRSS) algorithm to select nodes as probe stations by examining the distribution of sensor nodes and the fault points. We also proposed an adjusting rule for probing frequency after finding out the implicit information from the failure time and validating the Pareto principle.

The simulation indicates that the SRSS algorithm can prolong the lifetime of a network, and the proposed adjusting rule for probing frequency can reduce the number of useless probing packets. We also demonstrated that part of the Pareto principle could be used to detect faults in wireless sensor networks.

The rest of the paper is organized as follows. Section 2 reviews previous work on fault detection in wireless sensor networks and probe placement in common networks. In Section 3 we describe the details of SRSS and the adjusting rule for fault probing frequency. In Section 4 we present the simulation results. We conclude the paper in Section 5.

## 2. Related Work

The notion of fault detection, which was first introduced in the context of distributed control systems, often appears accompanied by fault isolation, and concerns itself with monitoring a system, identifying when a fault has occurred and pinpointing the type of fault and its location. When used in network management, it refers to the use of different metrics to collect symptoms of possible faults. There are a number of different fault detection approaches that have been studied. In this section, we summarize related works along two major lines: fault detection in wireless sensor networks, and probe selection strategy in traditional networks.

Sympathy [4] provided a centralized debugging technique to identify and localize the cause of failures in wireless sensor network applications. Nodes periodically send metrics back to a sink, which combines this information with passively-gathered metrics, analyzes the metrics to detect events, and identifies the spatiotemporal context of the events. The sink detects a failure if and only if some sink component does not receive sufficient packets. Lee *et al.* [5] proposed an adaptive policy-based management for wireless sensor networks, which provides a central manager to detect and localize faults by analyzing anomalies in wireless sensor network models. The central manager analyzes the topology map and the energy map to detect faults.

Suspicious (or failed) nodes can be identified via comparison of their sensor readings with neighbors’ median readings. Accordingly, Ding *et al.* [6] developed a localized algorithm to identify suspicious nodes whose sensor readings have large differences compared with the neighbors’ ones. This algorithm works for large sized wireless sensor networks. It assumes the probability of sensor faults needs to be small and each sensor node must be aware of its physical location. If half of the sensor neighbors are faulty, the algorithm cannot detect the faults as efficiently as expected, therefore, Chen *et al.* [7] proposed an improved approach, which does not require knowledge of the nodes’ physical position and no more than half the neighbor nodes.

Asim *et al.* [10] presented a new cellular architecture for fault management in wireless sensor networks. In this architecture, the network was divided into a virtual grid of cells, which can be considered as a special kind of clustering, and cells can be merged to form larger cells. One of the nodes in each cell is distinguished as the cell manager, and all cell managers form an upper level grid and the remaining nodes form a lower level grid. Based on above, they presented an energy efficient cellular approach to fault detection and recovery in wireless sensor networks [11].

Maitreya *et al.* [12–15] presented a series of heuristic based algorithms that incrementally select nodes which provide suitable locations to instant probe stations. They aimed to find a minimal set of probe station nodes so as to minimize the deployment cost, but they select fixed nodes as probe stations and assume that there are no failure probe stations in the network.

Jeswani *et al.* [16] and Liu *et al.* [17] considered the probe station selection problem in computer networks. They reduced the probe station selection problem to a minimum hitting set problem, and used different approximation algorithms to minimize the size of the hitting set, but they both assume that the routes do not change dynamically, a condition that cannot be satisfied in WSNs.

An intelligent probing approach is proposed in [18], where the authors formulate the probe selection problem as a constrained optimization problem—find the minimal subset of probes which has the ability to diagnose the problems of interest. The probes are selected by reasoning about the interactions between the probe paths. They implement algorithms which find near-optimal probe sets in linear time, but finding the optimal probe set is prohibitively expensive for large scale networks.

## 3. Simple Random Sampling Based Selecting Algorithm

In this study a probe station is a general sensor node which is selected through a specific strategy to perform three tasks: send out probing packets to its one-hop neighbors, receive feedback messages and send fault information to a cluster head. We mainly focus on how to select nodes as probe stations and how to determine the most appropriate probing frequency for fault detection. In this section, firstly we describe the network model and fault model, then we describe the details of our proposed selection algorithm.

### 3.1. Network Model and Fault Model

A variety of network environments must be considered in this study. As to the network topology, we study a large-scale sensor network that is deployed in a two-dimensional plane. In this work, we focus on an unstructured network case where the sensor nodes are randomly deployed. We assume that all sensor nodes are randomly deployed in a two dimensional rectangular area, and each node knows the coordinates (*x*, *y*) of its location. All nodes are numbered in sequence, and have the same communication and sensing range.

The sensors in a WSN are susceptible to a variety of faults, but all the different types of faults will eventually generate one or both of the following results: incorrect sensing data, or faulty communication. Whether the sensing data is correct or not is not our concern in network management. Therefore, similar to [8], the sensor nodes which generate incorrect sensing data but still could communicate with other nodes are treated as usable nodes, and only those sensor nodes with a permanent communication fault, or lack of power, are considered faulty nodes.

In general, a sensor node’s failure probability will change with time, and the longer the time a sensor node works the higher failure probability it will have. We can define the reliability *R_i_*(*t*) of a sensor node *s_i_* as the probability of not having a failure within the time interval (0, *t*) [21,22]:

(1) $$R_{i}\;(t)=e^{-\mathrm{at}}$$

where *α* is the failure rate of sensor node *s_i_*. The fault probability of node *s_i_* in time *t* is *F_i_*(*t*):

(2) $$F_{i}\;(t)=1-R_{i}\;(t)$$

### 3.2. Algorithm and Notation

In this section we first describe the Simple Random Sampling based probe station selection (SRSS) algorithm. SRSS is a random selecting algorithm which uses Simple Random Sampling (SRS) to select appropriate sensor nodes as probe stations to achieve the goal of distributing the energy load of the probe station evenly among the sensors in the network. The Pareto principle is used in SRSS to adjust the number of probe stations, LEACH [23] is used to cluster the network in each round. The algorithm is described as follows:

**Algorithm:** Simple Random Sampling based probe station selection for fault detection.

Initialize network.
Repeat
Cluster the wireless sensor network with LEACH.
Select elementary probe stations (in Section 3.3 and Section 3.4).
Select additional probe stations with Pareto rule (Section 3.5).
Begin/* fault detection
Send out probing packets.
Receive feed back messages.
Analyze feed back messages.
Determine which nodes are fault nodes.
If a node is determined as fault node the first time.
Then broadcast its id to other nodes.
End
End
Adjusting probing frequency(Section 3.6).
Until the network fails.

In addition, for convenience we give a definition and list the notation to be used in this paper.

*Definition*: **one-hop neighbor**: For ∀*s_i_*, *s*′ ∈ *S*, if the distance between *s_i_* and *s_i_*′ is less than or equal to *r*, then *s_i_*′ is a one-hop neighbor of *s_i_*. and *S*′(*i*) is the set which contains all the one-hop neighbors of *s_i_*.

**Table:** 

Notation	
*λ*	node density of the network
*N*(*A*),|*S*|	node number of the network
*A*	coverage region
‖*A*‖	area of coverage region
*f_a_*	area coverage
*c*	constant for connectivity function
*s_i_*	sensor node
*S*	set of sensor nodes
*s_i_*′	one-hop neighbors of node *s_i_*
*S*′(*i*)	set of one-hop neighbors of node *s_i_*
*r*	sensing radius
*Sp*	set of probe station nodes
*e_i_*	residual energy
*Sf*	set of fault nodes
*sf_i_*	fault sensor node
*Sc_k_*	set of nodes compose a cluster *k*
*R_i_*(*t*)	reliability of *s_i_* in time *t*
*F_i_(t)*	fault probability of *s_i_* in time *t*
*T_i_*	average lifetime
*α*	fault rate of sensor
*w*	time interval of sending probing packet
*fre*	probing frequency

### 3.3. The Number of Elementary Probe Stations

The number of probe stations depends on several factors, such as the coverage, the connectivity, the network topology and the relative costs of computation versus communication, *etc*. In this study we only discuss the coverage and the connectivity, the latter two parameters being set according to LEACH.

The coverage of a wireless sensor network is related to its deployment strategy. Under the assumption above, the locations of sensors can be modeled by a stationary two-dimensional Poisson point process [20]. We denote the density of the underlying Poisson point process as *λ*, which is measured by the number of sensor nodes per unit area. *N*(*A*) is the number of sensors located in a region *A*, which follows a Poisson distribution with parameter *λ*‖*A*‖P(), where ‖*A*‖ represents the area of the region *A*:

(3) $$P(N(A)=k)=\frac{e^{-\lambda \left\|A\right\|\;}{\left(\lambda \left\|A\right\|\right)}^{k}}{k!}$$

**Figure 1.**
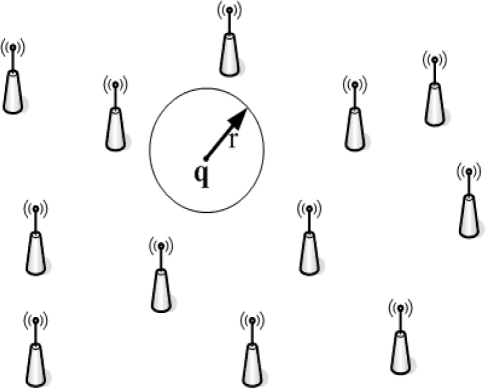
Determine area coverage *f_a_* for Poisson point process.

We then consider the situation shown in Figure 1, where a number of sensors are deployed uniformly with density *λ* and a selected point ***q*** are marked by a circle. This point is covered when there is at least one sensor present in the circle *A* of the radius *r* around ***q***. The probability to find at least one sensor in it is the ***area coverage*** [24]:

(4) $$f_{a}=P[N(A)\geq 1]\;=1-P[N(A)=0]\;\;=1-e^{{-\lambda \pi r}^{2}}$$

The area coverage shows the coverage condition of one wireless sensor network. Only under a certain area coverage, can a wireless sensor network work normally. To satisfy a prescribed area coverage *f_a_*, the required intensity *λ* of the Poisson point process is *λ* = –ln(1 – *f_a_*) / *πr*^2^. We can get the number of nodes to be placed in the sensing area from *N*(*A*) = λ* ‖*A*‖.

As to connectivity, there is a conclusion that the number of one-hop neighbors per node necessary for the network connectivity is a function *c*(ln *N*(*A*)) [25], where *c* is a constant. The asymptotic connectivity is achieved when *c* = 5.1774, with the critical value of *c* being close to 1. In any cases where *c* ≥ 1.5, the probability of connectedness increases to near 1 for a modest *N*(*A*) (e.g., *N*(*A*) ≈ 30). On the other hand, every *c*(ln *N*(*A*)) node could be considered as a subnet, in which all nodes can communicate with one special node. This special node is the probe station, which is different from the cluster head. *Sp*_1_ is the set of the elementary probe station of the network, the number of elements is determined by the number of clusters of the network:

(5) $$\left|{\mathrm{Sp}}_{1}\right|=\left\lceil N(A)/c*(\text{ln}\;N(A))\right\rceil \;=\left\lceil \lambda *\left\|A\right\|/(1.5*(\text{ln}(\lambda *\left\|A\right\|)))\right\rceil$$

As discussed above, the number of probe stations must be not too small and too large, which is actually determined by *f_a_* and *c*. In this study, we set *f_a_* = 0.999 for area coverage and *c* = 2.0 for asymptotic connectivity.

### 3.4. The Locations of Elementary Probe Stations

As discussed previously, if fixed nodes are chosen as probe stations throughout the network lifetime, those unlucky sensor nodes may die more quickly than others. Thus SRSS re-selects sensor nodes as probe stations in each round, so the sensor nodes are randomly selected as probe stations and this will not drain the batteries of the fixed sensors or the cluster-heads. Whether a node will be selected as a probe station or not depends on its residual energy and position.

To a given discrete distribution, if every individual unit in the population has the same chance of appearing in the sample, we can use Simple Random Sample method to sample individuals and the results still satisfy the same distribution with the population [26].

As discussed previously, if the nodes of a wireless sensor network are deployed randomly, then the locations of the nodes follow a Poisson distribution with parameters *λ* ‖*A*‖. The Poisson distribution is a discrete distribution, and every node in the network has the same chance to be selected as a probe station, therefore, by using the SRS method we can select appropriate nodes as probe stations, and the locations of the probe stations still follow a Poisson distribution [26]. For a given Poisson distribution:

(6) $$P(x=k)=P_{k}=e^{-\lambda }\frac{\lambda^{k}}{k!},\lambda >0$$

The SRS is:

(7) $$X_{F}=l,\;\;\mathrm{where}\;\sum_{i=0}^{l-1}\frac{\lambda^{i}}{i!}<\xi \cdot e^{\lambda }\leq \sum_{i=0}^{l}\frac{\lambda^{i}}{i!}$$

After selecting some nodes with their node number satisfy equation (7), we consider the residual energy of nodes as following:

***Rule 1***: ∃*s_i_* ∈ *S*, makes $e_{i}>=\frac{1}{\left|S^{\prime }(i)\right|}\sum_{j=1}^{\left|S^{\prime }(i)\right|}e_{j}$, where *e_i_*, *e_j_* is the residual energy of node, *s_i_*′ ∈ *S*′ (*i*) is the one-hop neighbors of *s_i_*, *S*′ (*i*) ⊂ *S*. To sum up, only the nodes which meet both Equation (7) and Rule 1 could be selected as probe station nodes.

### 3.5. The Additional Probe Stations

In most situations, the first fault node in a wireless sensor network will appear a certain number of rounds after the beginning, and, if a fault node appears in a cluster, the other healthy nodes in this cluster would have a higher probability of being out of order in the next several rounds. As time goes on, several nodes in a same cluster may be out of order simultaneously, so many fault messages will flood into the probe station node in this cluster. In this case, these clusters which contain much more nodes are likely to miss some fault messages and risk missing faults. Therefore we must select some additional probe stations in these clusters to prevent this from happening.

In this study, we use an evaluation function to determine the number of additional probe stations required. Before presenting the evaluation function, we discuss the Pareto principle first. The Pareto principle [19], also called the “20–80 rule”, summarizes this notion. The main idea is that a relatively small number of total faults or fault types will result in most of the poor quality in many different systems. The Pareto principle is used to concentrate efforts on the vital few instead of the trivial many. There are a number of examples of the Pareto principle in software engineering. Some of these have gained widespread acceptance, such as the notion that, in any given software system, most faults lie in a small proportion of the software modules. Schulmeyer and McManus [27] described how the principle supports defect identification, inspection, and applied statistical techniques. Fenton and Ohlsson [28] demonstrated that a small number of faults were responsible for a large number of failures, and used Pareto techniques to identify the most common types of faults found during pre-release testing and post-release testing. To the best of our knowledge, few works have applied the Pareto principle to the fault management problem in wireless sensor networks. In the following, we will investigate two related Pareto hypotheses:

Hypothesis 1: A small number of clusters contain most of the faults discovered.
Hypothesis 2: If a small number of clusters contain most of the faults discovered then this is simply because those areas constitute most of the sensor nodes.

The experiment is conducted 100 times using MATLAB. The node properties are set to follow MICA2. We now examine each of these in turn. When studying the fault management problem in wireless sensor networks, the node density and battery lifetime are the two important considerations [6–8]. In order to simulate different network environments, it is assumed that the sensor nodes have three types of communication range and two types of initial battery. Accordingly, the area of the monitoring field can be adjusted. The communication range, initial battery life and the matched monitoring area are randomly selected in the initial stage of the program. Table 1 shows the number of times of different network environments are adopted.

The simulation result shows that the fault conditions are different for the above six environments. The networks with the same communication range and different initial battery life have similar fault conditions. To the contrary, the fault conditions are very different when the networks have different communication ranges and same initial battery life. Thus, we find that the coverage will affect the global fault distribution.

#### (1) Hypothesis 1

**Figure 2.**
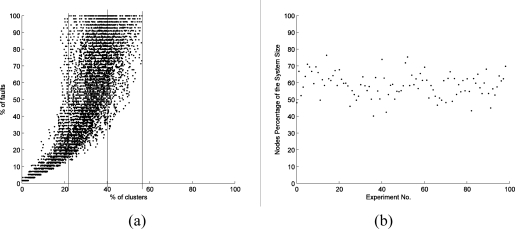
**(a)** Percentage of clusters *versus* percentage of faults. Each black dot denotes the correlations between the number of fault nodes and the number of clusters. **(b)** Nodes percentage of the system size. Each black dot denotes the proportion of the nodes which belong to those clusters that contain 80% fault nodes, to the total nodes of the wireless sensor network in one experiment.

Figure 2(a) shows the correlations between the number of fault nodes and the number of clusters. There are in total 5,561 faults in the experiments, and 4,631 of them are distributed in 40% of the clusters. That means that 40% of the clusters contain 83.3% of the faults. In extreme situations, some experiments suggest that no more than 30% of the clusters cover all the fault nodes. These results provide support for Hypothesis 1 and even suggest a specific Pareto distribution in the 40–80 area. Actually, almost all the results obtained in the experiments show that no more than 60% of the clusters contain 100% of the faults.

#### (2) Hypothesis 2

Since we found strong support for Hypothesis 1, it makes sense to test Hypothesis 2. To a certain extent, the Hypothesis 1 could be extended to the fact that the quite low percentage of the clusters contained all the faults just because these clusters contain most of the nodes.

As indicated in Figure 2(b), the clusters which have 80% of the faults (discussed in Hypothesis 1) contain 40–80 % of the nodes; the total number is 13,190 in the experiments. On average, they cover 132 nodes in each experiment, about 60 percent of the system size. Here we denote the number of nodes of a sensor network as system size. Therefore, in this study, we found no direct evidence to support this Hypothesis 2.

Hence, based on above analysis, we can conclude that most of the fault nodes are concentrated in some fields which occupy the relatively small percentage of the monitoring region. We will go even further to say that if a cluster has a fault node now then this cluster is likely to contain more faults nodes in the future. As a consequence, we have to select some additional sensor nodes as probe stations for this type of clusters.

Now we can start with the evaluation problem. To a given sensor set *S*, *sf_i_* is a fault sensor, ∃*Sf* ⊂ *S* and ∀*sf_i_* ∈ *Sf*. *Sc_k_* is the set that contains and only contains all member sensor nodes of cluster *k*, then we could select some additional probe stations for this cluster. These additional probe stations compose a set *Sp_k_*′ :

(8) $$\left|{{\mathrm{Sp}}^{\prime }}_{k}\right|=\left\lceil \left|{\mathrm{Sc}}_{k}\right|/\left|S\right|+\left|{\mathrm{Sc}}_{k}\cap \mathrm{Sf}\right|/\left|\mathrm{Sf}\right|\right\rceil$$

The additional probe stations are selected the same way as the elementary probe station nodes, but there is a difference that the candidates are limited to the member of this cluster. For the entire network, we must select $\sum_{k=1}^{\left|{\mathrm{Sp}}_{1}\right|}\left|{{\mathrm{Sp}}^{\prime }}_{k}\right|$ (|*Sp*_1_| is the number of elementary probe station nodes and the number of clusters of the network at the same time) additional probe stations. In this way, the clusters with more member nodes or fault nodes will have more probe stations than others.

### 3.6. Dynamic Adjusting Probing Frequency

In most situations, a wireless sensor network won’t have any faulty nodes in the initial several rounds. Thus probe stations send probing packets in each round, but cannot receive any useful feedback messages until the first fault node has appeared. In other words, all the probing work is useless during the period from the beginning to the time when the first fault nodes appear.

A way to considerably reduce this useless probing is to not send out the first probing packet until there is a failure in the network, this depends on the reliability of sensor node. As described earlier, *R_i_*(*t*) = *e*^−*at*^ is the probability of node *s_i_* not having a failure within the time interval (0,*t*). It is easy to see that the probability *R_i_*(*t*) means the probability of lifetime is larger than *t*_0_. Then, the average lifetime of the nodes is *T_i_*:

(9) $$T_{i}=\int_{0}^{\infty }R_{i}\;(t)\mathrm{dt}=\;\int_{0}^{\infty }e^{-\mathrm{at}}\mathrm{dt}=\frac{1}{\alpha }$$

The reliability of the entire network is *R*:

(10) $$R=\prod_{1}^{\left|S\right|}R_{i}\;(t)$$

Since the sensor nodes of the networks have the same *α*, the average lifetime (*T*) of the entire network must be:

(11) $$T=\int_{0}^{\infty }\prod_{1}^{\left|S\right|}R_{i}\;(t)\mathrm{dt}=\int_{0}^{\infty }e^{-|s|\mathrm{at}}\mathrm{dt}=\frac{1}{|s|\alpha }$$

where |*S*| is the number of nodes in the network. We can believe that there is no fault node before *T* in the sensor network. Thus we can reduce the probing packets until *T*, and cut down the number of useless probing packets. In this study, we decrease the probing frequency through dynamic adjustment of the probing frequency to reduce useless probing. A simple rule is described to show how to determine probing frequency:

(12) $$\mathrm{fre}=\{\begin{array}{ll} 1 & |\mathrm{Sd}|\geq 1\\ 1/w & \mathrm{other} \end{array}$$

In this way, we can reduce the useless energy consumption using the above rule, but the problem of missing faults must be considered when reducing the probing frequency. Because the probing packets are sent out every *w* round, any faults occurring during the interval between these probing packets and the next probing packets may be missed. For convenience we call this interval the “***probing interval***”. In this study, we adopt two measures to avoid this disadvantage:

- In any cases, the probe station nodes broadcast the probing packets to their one-hop neighbors. If the probe station node doesn’t receive feedback messages from one neighbor, this one could be the fault node, so even when the fault nodes occur in the time with no probing packets, they can still be detected out by the later probing packets. This however will result in some delay.
- Any sensor nodes of the network can store the messages of all fault nodes, and once a new fault node was detected out, the probe station broadcast the messages of this fault node to the neighbors. This way we could avoid broadcasting the repeat fault messages.

## 4. Simulation

Computer simulation was conducted to evaluate the performance of the proposed algorithm. Two different LEACH based algorithms are implemented, one is SRSS, and the other one is SIMPLE, which selects cluster heads as probe stations and sends out probing packets at a fixed frequency. MATLAB is used to perform all the simulations; both SRSS and SIMPLE were repeated 100 times.

### 4.1. Simulation Set-Up

The sensor nodes are deployed randomly in a 100 × 100 square region. Without loss of generality, we assume that the square region resides in the first quadrant such that the lower-left corner and the origin are co-located. For convenience we list the parameters to be used in this simulation in Table 2.

It is worth noting here that the number of the sensor nodes is 220, decided by the area of the monitoring region and the level of area coverage. We set *c* = *2.0* because when *c* ≥ *1.5* the probability of connectedness increases to near 1 [25], on average, per node has 10.8 one-hop neighbors in this 100 × 100 region. Since the faults occur in the ***probing interval*** will not be detected until the later probing packets are sent out, this will result in some delay. Too much delay will decrease the availability of the network, so we set the lower probing frequency to 0.5, *i.e.*, *w* = *2*. If the message of one fault is delayed more than two rounds, this fault is a missed fault.

We assume that all feedback messages would be received if the probe stations are working normally, and both faulty nodes and the nodes which are out of power are fault nodes. Fault detection rate (FDR) and lifetime are used to evaluate the performance of the SRSS. FDR is defined as the ratio of the number of fault nodes that have been detected, to the total fault nodes. We use a round number to measure the lifetime of node and network.

Note that the average lifetime of the entire network is *T* =1/(| *s* | *α*) = 1/220*10^−5^ = 454.5 according to Equation (11), and the value of *w* is 2. Hence, the probe station nodes send out probing packets every two rounds before the 455th round, and send out probing packets each round after the 455th round.

### 4.2. Distribution Character of Faults

**Figure 3.**
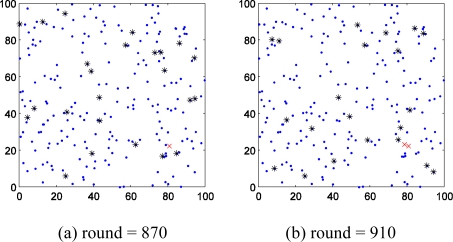

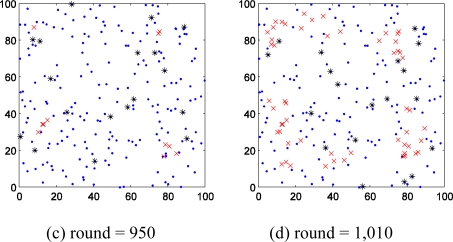
The distribution of fault nodes. * : cluster head node, • : simple nodes. × : fault node **(a)** the first fault node appeared; **(b)**&**(c)** several rounds later, more fault nodes appeared; **(d)** the number of fault nodes is so large that the network is useless.

We firstly study the spatial distribution character of faults. Figure 3(a–d) shows the spatial distribution of wireless sensor network nodes in one of the experiments. Figure 3(a) shows that the first fault point appeared in 870th round. Figure 3(b,c and d) shows that there are more and more fault points distribute in the monitor region, but the fault nodes could be grouped as several centralized parts. Thus we can draw the conclusion that the nodes near to fault node have higher probability to fail. This conclusion and Hypothesis (1) provide support to select additional probe stations.

In addition, the growth rate of fault nodes of wireless sensor network should be discussed too. In Figure 3(a) the first fault node appears in 870th round. In the following 40 round from the 870th, the number fault nodes increase to 2. But from 910th to 950th round, the same interval of 40 rounds, the number of fault node increases from 2 to 11. More surprising, the number rises to 55 only in the following 60 rounds.

**Figure 4.**
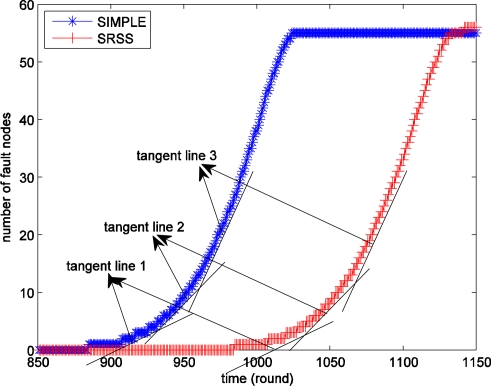
The average number of fault nodes in different time for the experiments.

We next study the information implied in Figure 4. The blue star line shows the number of the fault nodes at different times of the SIMPLE algorithm, which selects cluster head nodes as probe stations, and the red fork shows that of the wireless sensor network with SRSS. If the fault nodes increased at a fixed speed the lines must be straight and their slope is also fixed. Actually, the slopes of the lines are different. In order to see the result clearly, we have drawn some approximate tangent lines for the curves in Figure 4. As shown, the slope of tangent line 3 is larger than that of tangent line 2, and tangent line 2 is larger than tangent line 1. Therefore, we can believe that there is no fault node in the sensor network before a certain time and the number of fault nodes is increasing with time, but the more important thing is that the growth rate is also increasing with time. This provides important support to the use of dynamic adjusting of probing frequency.

### 4.3. Lifetime of Wireless Sensor Networks

Wireless sensor networks are power limited, and much work has been done to prolong the lifetime of the network. SRSS could reduce the energy consumption, evenly distributing the energy load among the sensors in the network to prolong the lifetime. In the simulation we suggest a wireless sensor network is useless (dead) if 25% of nodes have failed [24]. Here, the lifetime is the round number from the beginning to the time the network is dead.

**Figure 5.**
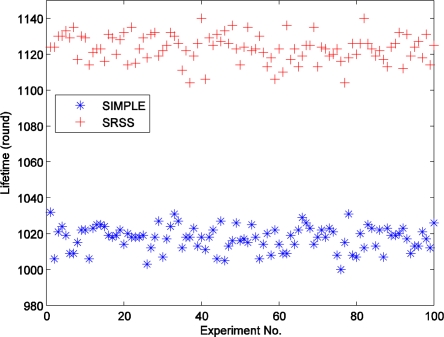
The lifetime of wireless sensor networks with different algorithms.

Figure 5 displays the different lifetimes of the two types of wireless sensor networks over 100 experiments. The figure shows that the SRSS results in a longer network lifetime than SIMPLE. Actually, the average lifetime of SIMPLE is 1,017.35, and that of SRSS is 1,123.32. The SRSS wireless sensor network thus has a 105.97 (10.4%) longer lifetime than SIMPLE. Therefore, we can believe that our approach could prolong the lifetime of wireless sensor network.

Additionally, Figure 5 also indicates that most of the lifetime of the algorithm with SRSS is between 1,100 and 1,140, and the one without SRSS is between 100 and 1,040. It means that both the two algorithms can achieve a relatively stable lifetime.

### 4.4. Fault Detection Rate

Another important thing we should consider when studying fault detection problems is the FDR (Fault Detection Rate). FDR is the proportion of faults have been detected by probe stations. We can get the FDR by dividing the number of nodes that have been detected by the total number of fault nodes.

**Figure 6.**
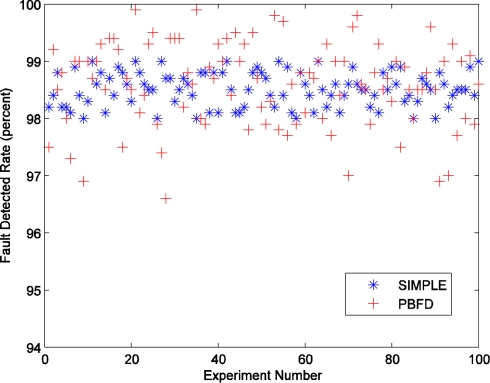
The fault detection rate of different algorithms.

Figure 6 describes the different FDR for every experiment with SIMPLE and SRSS. The figure shows that the blue stars fell on a relatively narrow area, but the red pluses are scattered over a larger area. This means that the FDR of the algorithm without SRSS is more centralized, but that of the algorithm with SRSS is decentralized. In a sense, this result may express that the SRSS would affect the FDR. The average FDR of SIMPLE algorithm is 98.6%, and that of SRSS algorithm is only decreased 0.1 points to 98.5%.

Although the FDR value of the SRSS algorithm is acceptable, we still need to find the factors which impact the FDR. The packet lost rate is not considered because that this ideal experiment environment would not lose any packets. Hence we track the experiments with the 10 lowest FDR of SRSS, and find that all these low FDR are caused by the failed probe station. If the probe station nodes happen to fail in the round they should send out probing packets, the faults will be detected at least two rounds later. This will lead to two more rounds of delay, and the faults would be missed. In extreme cases, one of the experiments with the 10 lowest FDR, the earlier several faults are missed because of the successively failed probe stations. The probing frequency is not adjusted to the normal value until one fault is detected. As a result, the FDR of this experiment is very low.

## 5. Conclusions

In this paper, we study the problem of probe station selection for fault detection in wireless sensor networks. We find that probe station selection strategy and fault probing frequency have a direct impact on the lifetime of a network. We next turned our attention to the probe station selection strategy and a rule for dynamically adjusting probing frequency. We also study the distribution of fault positions and the changing information on fault numbers.

Our results suggest that SRSS will evenly distribute the energy load among the sensors in the network and thus prolong the lifetime. Additionally, adjusting the probing frequency will reduce useless probing packets and further reduce wasted energy.

A certain delay is permitted in our algorithm to avoid missing faults, but this may limit the field of application of our algorithm. In the future we will attempt to design a constructive algorithm to solve this issue. Further, we find that the internal relationships between faults are also a meaningful problem in fault management in wireless sensor networks.

For now the mobility of a sensor is not considered, nor is the algorithm proposed in this paper suitable for a sensor network with mobile nodes. Although it is not difficult to extend the current algorithm to include sensor mobility, the extended algorithm does not yield good results, as the movement of nodes will change the distribution of the whole network and the locations of moved sensors will no longer follow a Poisson distribution. The idea of randomly selecting probing nodes is thus no longer applicable, hence forcing the design of a brand new algorithm to choose probe stations. Thus the mobility of a sensor will be taken into consideration in our future work.

## Figures and Tables

**Table 1.** The number of times of different network environments.

**Communication range**	**Initial battery life**	**Number of times**
5	0.5 joule	16
10	0.5 joule	18
20	0.5 joule	15
5	1 joule	15
10	1 joule	19
20	1 joule	17

**Table 2.** The simulation setting.

**Name**	**Value**	**Notes**
*r*	10	
*e_i_*	0.5 joule	The initial battery of the node.
*α*	1.0 × 10^−5^	The failure rate of a sensor node.
*f_a_*	0.999	99.9 percent of the area of monitoring region being covered.
*c*	2.0	
‖*A*‖	100×100	
*w*	2	
